# Supplementary material for: Lived experiences of women with low birth weight infants in the Solomon Islands: A descriptive qualitative study
Source: PLOS Glob Public Health. 2022 Dec 7;2(12):e0001008. doi: 10.1371/journal.pgph.0001008 (PMC10022132; doi:10.1371/journal.pgph.0001008)
Supplement: S1 Table — (DOCX) [file pgph.0001008.s001.docx]

**S1 Table. Women’s characteristics**

| **Characteristics** | **Frequency (n=18)** | **%** |
| --- | --- | --- |
| **Age (Years)** |  |  |
| <20 | 6 | 33 |
| 21-30 | 7 | 39 |
| 31-40 | 5 | 28 |
| **Area of residence** |  |  |
| Rural | 11 | 61 |
| Urban | 7 | 39 |
| **Ethnicity** |  |  |
| Melanesian | 16 | 88 |
| Micronesian | 1 | 6 |
| Polynesian | 1 | 6 |
| **Union (permanent relationship)** |  |  |
| In union | 15 | 83 |
| Not in union | 3 | 17 |
| **Education Status** |  |  |
| Primary school and lower | 5 | 28 |
| Secondary and above | 13 | 72 |
| **Employment status** |  |  |
| Unemployed | 16 | 89 |
| Employed | 2 | 11 |
| **Gravidity** |  |  |
| Primigravida | 8 | 44 |
| Multigravida | 10 | 66 |
