# Supplementary material for: Lived experiences of women with low birth weight infants in the Solomon Islands: A descriptive qualitative study
Source: PLOS Glob Public Health. 2022 Dec 7;2(12):e0001008. doi: 10.1371/journal.pgph.0001008 (PMC10022132; doi:10.1371/journal.pgph.0001008)
Supplement: S1 File — (DOCX) [file pgph.0001008.s002.docx]

**S1 File. COREQ checklist**

**Domains and items covered by the COREQ checklist**

| Topic | Item No. | Guide Questions/Description | Reported on Page No. |
| --- | --- | --- | --- |
| Domain 1: Research team and reflexivity |  |  |  |
| *Personal Characteristics* |  |  |  |
| Interviewer/facilitator | 1 | Which author/s conducted the interview or focus group? | Page 5 |
| Credentials | 2 | What were the researcher’s credentials? E.g., Ph.D., MD | Page 6 |
| Occupation | 3 | What was their occupation at the time of the study? | Page 6 |
| Gender | 4 | Was the researcher male or female? | Page 5, 19, 20 |
| Experience and training | 5 | What experience or training did the researcher have? | Page 20, 21 |
| *Relationship with participants* |  |  |  |
| Relationship established | 6 | Was a relationship established prior to study commencement? | Page 4-6 |
| Participant knowledge of the interviewer | 7 | What did the participants know about the researcher? e.g., personal goals, reasons for doing the research | Page 4-6  Supplementary File 2 |
| Interviewer characteristics | 8 | What characteristics were reported about the interviewer/facilitator? e.g., Bias, assumptions, reasons, and interests in the research topic | Page 19-20 |
| Domain 2: Study design |  |  |  |
| *Theoretical framework* |  |  |  |
| Methodological orientation and Theory | 9 | What methodological orientation was stated to underpin the study? e.g., grounded theory, discourse analysis, ethnography, phenomenology, content analysis | Page 5 |
| *Participant selection* |  |  |  |
| Sampling | 10 | How were participants selected? e.g., purposive, convenience, consecutive, snowball | Page 5 |
| Method of approach | 11 | How were participants approached? e.g., face-to-face, telephone, mail, email | Page 5-6 |
| Sample size | 12 | How many participants were in the study? | Page 5 |
| Non-participation | 13 | How many people refused to participate or dropped out? Reasons? | - |
| *Setting* |  |  |  |
| Setting of data collection | 14 | Where was the data collected? e.g., home, clinic, workplace | Page 5-6 |
| Presence of nonparticipants | 15 | Was anyone else present besides the participants and researchers? | Page 5-6 |
| Description of sample | 16 | What are the important characteristics of the sample? e.g., demographic data, date | Page 5-6  Table 1 |
| *Data collection* |  |  |  |
| Interview guide | 17 | Were questions, prompts, guides provided by the authors? Was it pilot tested? | Page 5-6  Supplementary file 2 |
| Repeat interviews | 18 | Were repeat interviews carried out? If yes, how many? | No |
| Audio/visual recording | 19 | Did the research use audio or visual recording to collect the data? | Page 7 Audio and transcribed |
| Field notes | 20 | Were field notes made during and/or after the interview or focus group? | Page 7 |
| Duration | 21 | What was the duration of the inter views or focus group? | Page 7 |
| Data saturation | 22 | Was data saturation discussed? | Page 5 |
| Transcripts returned | 23 | Were transcripts returned to participants for comment and/or correction? | NA |
| Topic | Item No. | Guide Questions/Description | Reported on Page No. |
| Domain 3: analysis and findings |  |  |  |
| *Data analysis* |  |  |  |
| Number of data coders | 24 | How many data coders coded the data? | Page 7 |
| Description of the coding tree | 25 | Did the authors provide a description of the coding tree? | Page 7 |
| Derivation of themes | 26 | Were themes identified in advance or derived from the data? | Page 8 |
| Software | 27 | What software, if applicable, was used to manage the data? | Page 7 |
| Participant checking | 28 | Did participants provide feedback on the findings? | NA |
| *Reporting* |  |  |  |
| Quotations presented | 29 | Were participant quotations presented to illustrate the themes/findings? Was each quotation identified? e.g., participant number | Page 8-15 |
| Data and findings consistent | 30 | Was there consistency between the data presented and the findings? | Page 8-15 |
| Clarity of major themes | 31 | Were major themes clearly presented in the findings? | Page 8 |
| Clarity of minor themes | 32 | Is there a description of diverse cases or a discussion of minor themes? | Page 8-15 |

Developed from: Tong A, Sainsbury P, Craig J. Consolidated criteria for reporting qualitative research (COREQ): a 32-item checklist for interviews and focus groups. *International Journal for Quality in Health Care*. 2007. Volume 19, Number 6: pp. 349 – 357
