# Supplementary material for: Lived experiences of women with low birth weight infants in the Solomon Islands: A descriptive qualitative study
Source: PLOS Glob Public Health. 2022 Dec 7;2(12):e0001008. doi: 10.1371/journal.pgph.0001008 (PMC10022132; doi:10.1371/journal.pgph.0001008)
Supplement: S2 File — (DOCX) [file pgph.0001008.s003.docx]

S2 File. Table of quotes not included in the results

| **Themes** | **Sub-themes** | **Other quotes on the themes that were not included in the results** |
| --- | --- | --- |
| Health issues | - Health problems women experienced during pregnancy - Women’s understanding of the causes of having LBW infants | - Yes, since the first month of my pregnancy, I was not well until I gave birth at 6 months. I had recurrent fevers, lethargy, and poor appetite. I could not see nurses or go to the clinic to check up because I was still at school. I was not sure what illness I had. I think I had malaria and had a preterm birth. I think malaria is a common health problem affecting many pregnant women. [P6, 20-year-old] - I was bleeding the entire pregnancy and that is the reason I have a small baby. I was referred from Kwaio in Malaita Provinces to NRH due to this. I delivered prematurely at 8 months. [P8, 23-year-old] - I had severe malaria during the first trimester. I was very sick and could not eat well. I have seen other women with pneumonia and flu during pregnancy. I was sick at six months and delivered the baby early. [P7, 30-year-old] - I have been having irregular periods, so I didn’t know that I was pregnant. I experienced no illness or dislike of food during pregnancy until 32 weeks I was sick with malaria. I saw Dr DB who gave me coartem 1st dose, 2nd and after dose 3rd dose, but I went into labour. They tried to stop the labour, but they could not, so I delivered my baby early.[P11, 34-year-old] |
| Substance use | - Substance use during pregnancy - Understanding the impact of substance use on pregnancy | - I also chewed betel nut and stopped due to the illness I had when I was pregnant and stop chewing until now. I have seen a lot of pregnant women who smoke tobacco and chew betel nut in my community. Tobacco smoke causes craving and addiction that and even in the middle of the night men and women will still go out to look for it. [P2, 39-year-old] - I chewed throughout my pregnancy. My mother told me to stop chewing but I did not stop. Now I am still chewing betel nut. I am not sure if betel nut is harmful to unborn babies during pregnancy. [P3, 16-year-old] - I often ate 3 betel nuts during pregnancy. I feel like to betel nut after my meal. It made me feel better after food. [P10,18-year-old] - I was a heavy betel nut chewer before pregnancy and reduced it during pregnancy. During pregnancy, I only chew when my mouth is sour. Betel nut helped reduce the bad taste. Yes, it will affect the baby because everything the mother consumes the baby will also receive. [P11, 34-year-old] |
| Diet and nutrition | - Knowledge of diet and nutrition - Dietary intake and nutrition quality - Social, cultural, and environmental impact on food supply and nutrition | - My diet is mostly comprised of rice, cassava, cabbage, beans, cucumber, and snake beans I normally had tea for breakfast, at lunch, I had rice and vegetable, and the same for dinner. Wind and rain destroy our vegetables. [P7, 30-year-old] - I normally ate kumara (sweet potatoes) green banana (plantain), rice, fish, and cassava. No, I did not receive information on diet and nutrition. Flood spoils our food garden causing a shortage of food. [P9, 25-year-old] - Our food supply was affected by strong wind, cyclones, and floods, which spoil our banana plants (plantains) and food garden. During heavy rains, they are usually very big floods which destroy our entire garden and we run out of food. Banana (plantain/green banana) is our staple food. [P5, 38-year-old] - In my culture (tribe) pregnant women should not eat eggs. It will cause sickness to babies e.g., boils on the head. [P1, 20-year-old] - We are not allowed to eat clam shells and some types of fish which I forgot their names. Bonito (Tuna) and salt fish are forbidden during pregnancy. [P10,18-year-old] |
| Domestic violence | - Presence of domestic violence in pregnancy - Understanding the impact of domestic violence on pregnancy and birth | - I personally experience so much domestic violence in my marriage, especially with my husband who often returns home drunk and started throwing plates, and spoons at me and all over the house. [P11, 34-year-old] - My husband beat me on my legs with a stick while I was 2 months Pregnant. He knew I was pregnant and did this only one time. Domestic violence can cause the baby to be born early or die before birth. (Prematurity or stillbirth) [P12, 18-year-old] - My husband beat me countless times and cause me injury and bleeding. It was a terrifying experience. I sustained major and minor injuries. Sometimes he pushes me off and fell onto the concrete floor. He stopped a while ago before the last pregnancy domestic violence can cause stillbirth. Some men can be uncontrollably angry ended up beating up on their wives’ backs or tammy which can lead to death in the unborn child (stillbirth). [P13, 25-year-old, rural] - He slapped me in my last pregnancy. He did not kick me. I was affected emotionally. He apologized to me before delivery. I believe this affected my pregnancy. [P17, 35-year-old] |
| Environmental conditions | - Dwellings and overcrowding - Quality of water supply and toilet facilities | - I lived in a sago palm-built 2-bedroom house with 6 people living there. I shared a room with my sister. [P9, 25-year-old] - We lived in a thatched sago palm 3-bedroom house. There are three of us there our couple and his younger sister who still goes to school. There are plenty of rats and dogs around. [P14,19-year-old] - We do not have a proper toilet. We just use the bush as a toilet. If it were in Wagina, we would use the seaside but here in Guadalcanal inland, we just use the bush (chuckled). This is not right. I always complain to my husband about this. The water supply pipes were destroyed. During sunny days the borehole dries up, so we have to go a long distance to get water. [P18, 18-year-old] - We do not have a proper water supply. We usually go down the hill to the main road to a stream few metres down. We live on Marble Street near the public phone. The water spring is close to the drain on the side of the road. [P10,18-year-old] |
| Antenatal Care | - Access to antenatal health care service - Quality of antenatal care and services | - My local nearby clinic is Fox Bay clinic of west Guadalcanal. We must paddle in a dugout canoe for 30 minutes to get there. [P16, 32-year-old] - Nurses must give treatment to mothers; accordingly, I think Nurses should improve their service. Government should provide proper instruments and medicine for nurses to use in antenatal clinics. [P6, 20-year-old] - It’s a long way to go to the clinic. It is very tiring. I started my trip at 8 am and reached the clinic at 11 am. I would wait for hours before the nurse sees me. The nurse work very slow and there were piles of antenatal cards from the many mothers waiting. There are so many pregnant women. I would finally see the nurse at 3 pm. I retired back and would arrive back home at dusk (6pm) This was really a big challenge for me. [P18, 18-year-old] - My home village is far from the clinic. We have to walk up and down the hill for 2 and a half hours to get to the clinic. I was only supplied with tonic (ferrous sulphite tablets) which I took daily. The malaria medicine and the medicine for worms were out of stock. I am satisfied with the nurses’ advice and counselling. [P17, 35-year-old] |
