# Supplementary material for: Lived experiences of women with low birth weight infants in the Solomon Islands: A descriptive qualitative study
Source: PLOS Glob Public Health. 2022 Dec 7;2(12):e0001008. doi: 10.1371/journal.pgph.0001008 (PMC10022132; doi:10.1371/journal.pgph.0001008)
Supplement: Attachment — Submitted filename: Response to reviwers.docx [file pgph.0001008.s004.docx]

Response to the Editor and Reviewers
Lived experiences of women with low birth weight infants in the Solomon Islands: a descriptive qualitative study (PGPH-D-22-01214)

**EDITORS COMMENTS**

Please include the following items when submitting your revised manuscript:

a) A rebuttal letter that responds to each point raised by the editor and reviewer(s). You should upload this letter as a separate file labeled 'Response to Reviewers.'

**Response:** We have addressed all comments point-by-point and uploaded the document as a separate file.

b) A marked-up copy of your manuscript that highlights changes made to the original version. You should upload this as a separate file labeled 'Revised Manuscript with Track Changes.

**Response:** As requested, we have provided a marked-up version reflecting our changes addressing comments from the editor and reviewers labelled as “manuscript review copy.”

c) An unmarked version of your revised paper without tracked changes. You should upload this as a separate file labeled 'Manuscript'.

**Response:** A clean copy of the edited manuscript is attached and labelled as “manuscript clean copy.”

**Journal Requirements**

a) Note from Staff Editor Katrien Janin (kjanin@plos.org): PLOS considers qualitative and mixed-methods studies for publication. We recommend that authors use the COREQ checklist, or other relevant checklists listed by the Equator Network, such as the SRQR, to ensure complete reporting (https://journals.plos.org/globalpublichealth/s/submission-guidelines#loc-qualitative-research).

**Response:** We were guided by the COREQ checklist for reporting the study and we have presented it as supplementary material (i.e., Supplementary file 1)

In general, we would expect qualitative studies to include the following:

b) defined objectives or research questions;

**Response:** The research aim is stated on page 4 paragraph 3 of the manuscript as follows:

*“This study aimed to understand the lived experience during the most recent pregnancy of women in the Solomon Islands who gave birth to LBW infants by exploring and describing their personal (socio-demographic and health), behavioural, social and environmental context during their pregnancy.“*

c) description of the sampling strategy, including rationale for the recruitment method, participant inclusion/exclusion criteria and the number of participants recruited;

**Response:** Sampling strategies and rationale for the methods are stated on page 5 paragraph 1 of the manuscript:

“*We employed a descriptive qualitative study using purposive sampling to recruit women with LBW infants* *to participate in one-on-one in-depth interviews. This methodology was deemed appropriate to explore women’s personal stories and experiences during* *their recent pregnancies* *resulting in LBW babies [35,36]”*

We have also described the number of participants and inclusion and exclusion criteria on page 5 paragraph 2 of the manuscript:

*“Eighteen postnatal women aged 15 to 39 years were recruited ….The women were required to have given birth to LBW infants weighing less than 2500 grams, irrespective of gestational age, within the last week before recruitment. It was also a requirement that the health of the mother and infant was stable. Women were required to be fluent in Pidgin English, the Solomon Islands lingua franca.”*

d) detailed reporting of the data collection procedures;

**Response:** Details of the data collection procedure are stated on page 5 paragraph 3 to page 7 paragraph 1 in the revised manuscript under *the* ***“Data collection”*** subsection.

e) data analysis procedures described in sufficient detail to enable replication;

**Response:** Details of the data analysis procedures are documented on page 7 paragraph 2 of the manuscript. *“The translated and transcribed data were uploaded into NVivo and then analysed using thematic analysis [38, 39]. This involved repeatedly reading through each interview to become immersed and familiar with its content and annotating meanings arising from the data; generating succinct labels (codes) to identify essential features of the data; collating data to identify significant broader patterns of meaning and potential themes; reviewing themes against the dataset to ensure they told the data’s story; defining and naming themes; and weaving together the analytic narrative with quotes to illuminate themes [38, 39]. For validity and reliability, co-authors (JJ and GAT) who are experienced in qualitative research provided guidance and reviewed the generated themes with the lead author (LSK), leading to a consensus on the final themes.”*

f) a discussion of potential sources of bias; and a discussion of limitations.

**Response:** Potential sources of bias are documented on page 19 paragraph 3 of the manuscript under the section titled ***“Strengths and Limitations”***

Please amend your detailed Financial Disclosure statement. This is published with the article. It must therefore be completed in full sentences and contain the exact wording you wish to be published.

a) **Response:** The Financial Disclosure statement is as follows: *“GP was supported by funding from the National Health and Medical Research Council Project and Investigator Grants #1099655 and #1173991, and the Research Council of Norway through its Centres of Excellence funding scheme #262700. GAT was supported by funding from the National Health and Medical Research Council Investigator Grant #1195716. LSK is a recipient of the Australia Award scholarship from the Department of Foreign Affairs”*
b) State the initials, alongside each funding source, of each author to receive each grant.

**Response:** Funding was amended in the editorial manager portal, and we ensured that we are consistent in our funding information section as on page 22 in the revised manuscript.

c) State what role the funders took in the study. If the funders had no role in your study, please state: “The funders had no role in study design, data collection and analysis, decision to publish, or preparation of the manuscript.” If you did not receive any funding for this study, please simply state: “The authors received no specific funding for this work.”

**Response:** We have updated our funding information and we indicated that the funders had no role in study design, data collection, and analysis, decision to publish, or preparation of the manuscript. The funding statement on page 22 paragraph 2 of the manuscript states *“GP was supported by funding from the National Health and Medical Research Council Project and Investigator Grants #1099655 and #1173991, and the Research Council of Norway through its Centres of Excellence funding scheme #262700. GAT was supported by funding from the National Health and Medical Research Council Investigator Grant #1195716. LSK is a recipient of the Australia Award scholarship from the Department of Foreign Affairs.”*

d) We have noticed that you have uploaded Supporting Information files, but you have not included a list of legends. Please add a full list of legends for your Supporting Information files after the references list.

**Response:** We have now included a list of legends after the conclusion paragraph on page 20 entitled ***“supporting information***

*Table 1. Women’s characteristics*

*Supplementary file 1. COREQ checklist*

*Supplementary file 2. Table of quotes not included”*

e) Please review your reference list to ensure that it is complete and correct. If you have cited papers that have been retracted, please include the rationale for doing so in the manuscript text or remove these references and replace them with relevant current references. Any changes to the reference list should be mentioned in the rebuttal letter that accompanies your revised manuscript. If you need to cite a retracted article, indicate the article’s retracted status in the References list and also include a citation and full reference for the retraction notice.

**Response:** We have reviewed all our references and ensured that complete information has been provided. All web links connecting references to online sources were checked. Reference 14 (Gibson et al 2015) and 20 (WASH 2015) online links to articles were retracted, and the correct link was now added.

**COMMENT FROM REVIEWERS**

**REVIEWER #1: 1. Summary of the Research**

Research question, claims, and conclusions of the study
Thank you for providing this opportunity to read and provide feedback on this article. This research article reports on the lived experience of a purposive sample of 18 Solomon Islands women who had recently given birth to a baby of low birth weight, through the usage of a qualitative descriptive methodology. The interview protocol was reviewed by experts and administered in the native language to the women participants. Due to travel restrictions, interviews were conducted via telephone. Recordings were translated into English and NVivo used for analysis. Six thematic areas were identified; health issues, substance use, diet and nutrition, domestic violence, environmental conditions and antenatal care. The authors’ findings submit that the women were exposed to different personal, behavioral, social and environmental risk factors that can affect birth outcomes with specific mention of LBW

Context for how this research fits within the existing literature

The authors make comparison to other published studies in other regions of the world such as Asia and the USA and contextualizes their findings with other published articles on the thematic area in the region where possible (example of PNG). The authors utilize reports produced by the Solomon Islands to contextualize their findings such as reports on domestic violence. They seek to fulfill a gap in the published literature on the lived experiences of women in the Solomon Islands during their pregnancy.

Discuss the manuscripts strengths and weaknesses and your overall recommendations
This is very interesting report that sheds light on the experiences of pregnant women in the Solomon Islands. The authors provide a sound rationale for the methodology chosen and the approach to data collection and analysis. The inability to conduct the interviews in person removed the ability to respond to facial expressions and non-verbal cues. With some revision, the report would be a lovely addition to the body of literature in this space.

The recommended changes are:

a) include a section on setting in the background or methodology to assist the reader to better understand the situation in the Solomon Islands – the location, development status, ratio of health workers to population, percentage of births that are LBW etc.

**Response:** The location and development status of the Solomon Islands and the percentage of LBW's are stated on pages 3-4 of the revised manuscript. We have now added, the ratio of health workers to population and a comparison to the Asia Pacific on page 3 paragraph 2 of the manuscript, as follows:

“*The number of health workers per population density is lower than that of most countries in the Asia Pacific region. Reports show there were approximately 19 doctors, 145 nurses, and midwives per 100 000 of the population. [17, 18].*

b) Include a detailed discussion that compares and situates that the thematic areas within the data available on the Solomon Islands.

**Response:** Our comprehensive review of the literature identified limited local data that corresponded to the thematic areas. In the discussion section, we have already discussed the thematic areas in relation to several relevant studies that are available. For example, we have identified two local studies on domestic violence during pregnancy (23,64), four nutrition studies (50, 54, 55,57), one for sexually transmitted infections (48), and two relating to perinatal health (26,70). There were no local studies on the other identified themes.

c) Consider including the statistics referred to instead of only including the citation, to provide the reader with specific information.

**Response:** We have now included statistics on the ratio of health workers to population have been added on page 3 paragraph 2 as: *“The number of health workers per population density is lower than that of most countries in the Asia Pacific region. In 2012 there were 19 doctors, 145 nurses, and midwives per 100 000 of the population [17]. However, based on the latest report, the numbers have improved to 11.2 nurses and doctors per 1000 people or 1120 doctors and nurses per 100 000 population [18].”*

Statistics on the prevalence of LBW in countries of Melanesia (PNG and Vanuatu) have now been expanded on page 4 paragraph 2 as follows: *“The Solomon Islands has a prevalence for LBW of 10%, which places it within the minimal range compared to South-East Asian countries and Melanesian countries of the Pacific such as PNG and Vanuatu (between 10-17% prevalence) [2, 25-27]. However, this rate is elevated compared to some countries in the Polynesia region, with Tonga, Samoa and Tuvalu reporting LBW of well below 7% [2, 27].”*

The Solomon Islands is a Melanesian country culturally but is compared to data from countries in the Polynesia region, perhaps a comparison can also be made to other Melanesian countries.

**Response:** We agree with the reviewer. Due to the paucity of evidence in the region we thought we could compare our results with Melanesian countries, as seen on page 4 paragraph 2:

*“The Solomon Islands has a prevalence for LBW of 10%, which places it within the minimal range compared to South-East Asian countries and Melanesian countries of the Pacific such as PNG and Vanuatu (between 10-17% prevalence) [2, 25-27]* ”

d) Introduce each of the acronyms

**Response:** We have now ensured that we introduced all the acronyms in our first use in the paper.

e) Review the grammar (there were several mismatches between the noun and verb).

**Response**: We have now thoroughly reviewed the manuscript for grammatical errors.

f) Provide the range of the length of time of interviews instead of only the average

**Response:** The interview times ranged from 30 to 70 minutes. This has been added to page 7 paragraph 1 of the manuscript as follows:

*“**The interviews ranged from 30 to 70 minutes, with an average time of 54 minutes.“*

g) Format to highlight long quotes.

**Response:** Thank you for your suggestion, but the long quotes are presented in the format that most papers in PLOS Global health journal are presented.

h) The usage of the words, many, several, most, some, should be companied by a number.

**Response:**  As requested, we have added numbers to the results. See track changes throughout the results section of the manuscript.

**REVIEWER #2: Summary of review:**

Overall, this paper contributes important qualitative information on LBW in a region that has little data. There were grammatical (including run-on sentences) and spacing errors, so authors should perform a close edit of the paper overall. The authors used the COREQ framework, which ensured that the methods and reporting were high quality. The results are described well with illuminating quotes and are important findings that could direct future research on the topic. The discussion was also clear and could benefit if the authors could expand on other public health policies in the country related to the themes they identified.

Comment

a) To meet the open data sharing requirement of the journal, authors could also produce a table with other quotes on the themes that were not included in the results.

**Response:**

We have now provided lists of all included quotes/participants reflections provided in our themes and sub-themes as Supplementary file 3

**Abstract**
b) Should be comma: “qualitative descriptive”

**Response:**  Thank you for the suggestion. This has been addressed.

c) Could be nice to remind the reader what the themes were describing in this sentence under “Findings.”

**Response:**
We have now reminded the reader what the themes are describing, as follows:

*“We identified six themes reported as being related to LBW: health issues, diet and nutrition, substance use, domestic violence, environmental conditions and antenatal care”*

**Introduction**
a) Sometimes “socio-demographic” and other times “sociodemographic” so please choose one way of spelling.

**Response:** Thank you. We have updated this throughout the manuscript using *“socio-demographic”*

b) spacing error between “such as” and “tobacco”

**Response:** Thanks for noting this and we have now corrected this

c) another spacing error, “poverty[14]”

**Response:** spacing was corrected

d) Could state which countries are below 7% LBW for readers

**Response:** The sentence has been amended on page 4 paragraph 2 as follows.

“*However, this rate is elevated compared to some countries in the Polynesia region of the Pacific, with Tonga, Samoa and Tuvalu reporting LBW of well below 7% [2,27].”*

e) “Furthermore, no studies have”

**Response:**  Corrected (page 4 paragraph 3)

**Methods**
a) “The recruitment of participants was halted when data saturation was reflected in no new information emerging.” How did the authors define saturation and know when they reached it?

**Response:** We have stated on page 5 paragraph 2 of the manuscript that saturation was reached or defined when no new information emerged.

b) The interview schedule was assessed by an expert panel. Could the authors please describe this in further detail, such as the qualifications of who was on the panel.

**Response:** The sentence has been amended on page 6 paragraph 1 as follows:

*“The interview schedule was assessed for face and content validity by an expert panel who have PhDs and expertise in clinical and public health research* *”*

c) The interview questions asked about”, sound colloquial and makes sentences difficult to read.

**Response:** The sentence has been amended for clarity on page 6, paragraph 1 as follows:

*“**We asked the women about their health, health behaviours, social and environmental conditions, access to antenatal care and their understanding of risk factors during pregnancy that may have contributed to having a LBW infant”*

d) Could the authors be more specific on how they used bracketing as a method? What were the preconceived notions?

**Response:** Bracketing was undertaken through dialogue between the researchers to identify potential preconceived notions that might influence the data collection and analysis. From this, a list of preconceived notions was written on a memo as a guide to refer to during the data collection and analysis. Some examples of preconceived notions were “most women know that betel nut is harmful for pregnancy, or the existence of food taboo during pregnancy." We ensured that women subjectively tell their stories, and these stories are not tainted with our prior knowledge and experiences.

**Results**
Quotes were integrated nicely into the reporting of the results and painted a vivid picture of the experiences of the women in this study

**Response:** Thank you

**Discussion**
a) Is there any scholarly work done on policy or public health education in the Solomon Islands on themes identified in this paper, such as substance use during pregnancy or domestic violence? This could be an important discussion point of the applications of this research. Expanded discussion on policies or education like the authors did of the Family Protection Act, could provide important political background and highlight potential areas to target for interventions.

**Response:** Health policy and public health education in the Solomon Islands on all the identified themes are not entirely available. As the reviewer noted, there is public health policy for family protection which has been cited as reference [68]

In the revised version, we have now discussed health policies on nutrition, water and sanitation and health care services.

Statement relating to nutrition policy has been added to page 17 paragraph 2, as follows.

*"This is despite the introduction of the National Food Security, Food Safety and Nutrition Policy (2019-23) by the Ministry of Health and Medical Services (MHMS) [57]. Priority policy areas included better nutrition for vulnerable populations (e.g., women and children), strengthening of the food supply chain, awareness of safe and healthy food choices and promotion of health and nutrition.* *To date, it remains uncertain whether this has been well implemented at a consumer level.”*

Statement relating to safe water and sanitation has been added to page 19 paragraph 2 as follows:

*“Poor water and sanitation remain pervasive in rural and urban areas of the Solomon Islands, even though a National Water and Sanitation Policy 12-year implementation plan was introduced in 2017 [73].”*

Statement related to health care has been added to page 18 paragraph 2 as follows:

*“The MHMS Role Delineation Policy underpins and safeguards affordable and accessible health care for its citizens, yet access to health care services remains a challenge [69]”*

b) In the limitations, although it could be a perceived as a strength to have the interviewer be a health professional, have the authors considered whether this was also a limitation?

**Response:**. Using female health professionals as the interviewers had several advantages. We also appreciate the view that use of health professionals as interviewers may also introduce social desirability bias and we have now included additional discussion in the limitations accordingly as follows:

“*Using a highly trained health professional as the interviewer can also be seen as a limitation as they may presume a greater level of participant knowledge than non-health professionals. Health professionals may themselves be influenced by aspects of care based on their prior experience which governs the dialogue and interpretation. Participants may be less well-included to open up to trained health professionals who maintain their professional identity throughout the interview process compared to non-health professionals who are perceived as peers.”*

c) Could there be a reporting bias is interviewees give answers that they think the doctor would like to hear? It doesn’t appear so, given that the participant spoke freely about some unhealthy behaviours of substance abuse during pregnancy, but it could be nice for authors to explore the provider/interviewee dynamic more.

**Response:** We agree with the reviewer regarding the potential of social desirability bias, and we have highlighted this bias in the limitation section.
